# Supplementary material for: Mitochondrial Transfer to Host Cells from Ex Vivo Expanded Donor Hematopoietic Stem Cells
Source: Cells. 2023 May 25;12(11):1473. doi: 10.3390/cells12111473 (PMC10252267; doi:10.3390/cells12111473)
Supplement: Supplementary file 1 [file cells-12-01473-s001.zip › cells-2378664-supplementary.pdf]

Supplementary Table S1

Antibody		Fluorophore		Clone
**Stem and Progenitor Cell Panel**				
**Antibody**		**Fluorophore**		**Clone**
Lineage		PerCP-Cy5.5		
	Anti-Ter119			Ter-119
	Anti-Gr1			RB6-8C5
	Anti-B220			RA3-6B2
	Anti-CD3e			145-2C11
Anti-CD16/32 (Fc γR)		BV786		2.4G2
Anti-CD150 (SLAM)		BV605		TC15-12F12.2
Anti-Flt3		BV421		CD135
Anti-CD48		PE-Cy7		104
Anti-cKit		PE-Cy5		2B8
Anti-Sca1		PE-CF594		D7
Anti-CD34		PE		RAM34
Anti-CD41		BUV395		MWReg30
**BM Mature Cell Panel**				
**Antibody**		**Fluorophore**		**Clone**
Anti-CD3e		PerCP-Cy5.5		145-2C11
Anti-CD45		APC-Cy7		30-F11
Anti-CD11b		AF700		M1/70
Anti-CD206		AF647		C068C2
Anti-Ly6G		BV786		1A8
Anti-B220		BV605		RA3-6B2
Anti-Ly6c		PE-Cy7		AL-21
Anti-CD4		PE-Cy5		RM4-5
Anti-F4/80		PE-Texas Red		BM8
Anti-CD8a		BUV395		53-6.7
**HSC Sort Panel**				
**Antibody**		**Fluorophore**		**Clone**
Lineage		Biotin (primary)		
	Anti-Ter119	APC-Cy7-Streptavidin (secondary)		Ter-119
	Anti-Gr1			RB6-8C5
	Anti-B220			RA3-6B2
	Anti-CD3e			145-2C11
Anti-cKit		APC		2B8
Anti-CD150 (SLAM)		BV785		TC15-12F12.2
Anti-Sca1		PE-CF594		D7
Anti-CD11b		APC-Cy7		M1/70
**HSC in Vitro Quality Check Panel**				
**Antibody**		**Fluorophore**		**Clone**
Anti-CD34		FITC		
Lineage		Biotin (primary)		
	Anti-Ter119	APC-Cy7-Streptavidin (secondary)		Ter-119
	Anti-Gr1			RB6-8C5
	Anti-B220			RA3-6B2
	Anti-CD3e			145-2C11
Anti-cKit		APC		2B8
Anti-CD4		PE-Cy5		RM4-5
Anti-CD150 (SLAM)		BV785		TC15-12F12.2
Anti-CD48		PE-Cy7		104
Anti-Sca1		PE-CF594		D7

Supplementary Table S1. List of antibodies in each panel
The fluorophore and clone information of each anti-mouse antibody was provided.
